# Insulin resistance in Iraqi women with idiopathic hirsutism in Najaf City: A case–control study

**DOI:** 10.5339/qmj.2024.72

**Published:** 2024-12-21

**Authors:** Maysam Alaasam, Jalal Al-bdairi, Raffat Abboodi

**Affiliations:** ^1^College of Medicine, University of Kufa, Kufa, Iraq; ^2^Endocrine and Diabetes Center, Najaf, Iraq; ^3^Alnajaf Teaching Hospital, Najaf, Iraq *Correspondence: Maysam Alaasam. Email: maysam.alaasam@gmail.com

**Keywords:** Idiopathic hirsutism, insulin resistance, HOMA-IR, Iraq

## Abstract

**Background:** Hirsutism is a common endocrine problem with high prevalence in Iraqi women. Polycystic ovarian syndrome and idiopathic hirsutism (IH) are the most common etiology of this disorder. There is a clear association between insulin resistance and polycystic ovarian syndrome. However, there is insufficient data on the relationship between insulin resistance and IH.

**Aim:** The aim of this study was to determine whether Iraqi women with IH have insulin resistance.

**Methods:** The study included two groups: 60 Iraqi women with IH and 60 women without hirsutism as a control group. A physical examination, a medical history, and the patient's age and BMI were collected. Blood samples were collected for hormone analysis, including insulin, follicle-stimulating hormone, luteinizing hormone, prolactin, dehydroepiandrosterone sulfate, 17-hydroxyprogesterone, and free testosterone. To evaluate insulin resistance in both groups, the homeostasis model assessment-insulin resistance (HOMA-IR) and the quantitative insulin sensitivity check index (QUICKI) were used in addition to the Matsuda index.

**Results:** Basal insulin was higher in the IH group (9.5 ± 0.2 mIU/L) than in the control group (4.8 ± 3.7 mIU/L), with a *p* value of < 0.0001. HOMA-IR was higher in the IH group (2.3 ± 4.1 μU mg), while in the control group, it was (0.8 ± 1.2 μU mg), with a *p* value of 0.007. There was a significant difference in the QUICKI, which was lower in the IH group (0.31 ± 0.2 μU^-1^/mg) than in the control group (0.45 ± 0.1 μU^-1^/mg), with a *p* value of < 0.0001. The insulin sensitivity index (Matsuda) was significantly lower in the IH group (3.1 ± 0.4) than in the control group (7.8 ± 1.3), with a *p* value of < 0.0001.

**Conclusion:** According to the results of this study, Iraqi women with IH have insulin resistance and higher basal insulin levels.

## INTRODUCTION

Hirsutism is the excessive growth of coarse terminal hair in women in a male-like pattern.^1^ It is important to distinguish hirsutism from hypertrichosis, which is excessive hair growth anywhere on the body in either males or females.^1^

Traditionally, hirsutism has been understood to be a sign of elevated androgen levels in females, either as a result of an ovarian or adrenal disease.^2^

Polycystic ovarian syndrome (PCOS) and ovarian tumors are the most common causes of overproduction of androgens in the ovaries.^3^ In contrast, Cushing's syndrome, androgen-producing tumors, and congenital adrenal hyperplasia (CAH) represent the most common adrenal causes.^4,5^ Hyperprolactinemia is another factor that contributes to hirsutism, as it increases the levels of adrenal dehydroepiandrosterone sulfate (DHEAS).^6^ Among the different causes of hyperandrogenism that lead to hirsutism, PCOS remains the most common etiology.^7^ PCOS is a complex syndrome of unknown etiology characterized by hyperandrogenism and chronic anovulation.^8^ Hyperinsulinism with insulin resistance is a well-known feature of PCOS that leads to the activation of pituitary insulin receptors that produce luteinizing hormone (LH) and increase androgen secretion by the ovary. Additionally, it can limit the formation of hepatic SHBG (sex hormone-binding globulin) and increase free testosterone levels.^8^ Hirsutism in PCOS is caused by excess androgen production from the ovaries and increased sensitivity of the pilosebaceous unit to androgens and insulin.^9^ Additionally, according to Itami et al., androgen stimulates the growth of hair follicles in association with insulin and insulin-like growth factors.^10^

Twenty percent of patients with hirsutism suffer from idiopathic hirsutism (IH), which is characterized by normal ovarian and adrenal function.^11^ The pathogenesis of hirsutism in these women is most likely due to disorders in peripheral androgen activity and increased local activity of the 5α-reductase enzyme.^11^ IH is the second most common etiology of hirsutism, after PCOS.^12^ The association between insulin resistance and hyperandrogenism is well established. However, the question of insulin resistance in IH is rarely studied, especially in Iraq.

Hirsutism is a common problem with high prevalence in Iraqi women, according to a study published in 1992, which also showed that Iraqi women have different hair distribution patterns compared to people of Welsh origin.^13^ There have been numerous studies of hirsutism in Iraqi women, but all have focused on the etiology, pathophysiology, and prevalence of the disease in the region. The association between IH and insulin resistance needed to be explored.

Therefore, the aim of this study was to determine whether Iraqi women with IH have hyperinsulinemia and insulin resistance.

## MATERIALS AND METHODS

### Study design

A case–control study was conducted in Alnajaf, Iraq between February 2022 and October 2023.

A total of 60 Iraqi women with IH and 60 healthy matched control groups were recruited from the Endocrine and Diabetes Center, Department of Internal Medicine at Alsadar Medical City, and Alzahraa Teaching Hospital. All participants completed the study.

### Ethical approval

The objective of the study was explained and informed consent was obtained from all participants.

The present study followed the Declaration of Helsinki (revised in 2013). In addition, it was approved by the Ethics Committee of the Medical College, Kufa University, Iraq.

### Participants and procedures

A detailed medical history was collected from all patients. The medical history includes the age of onset, the rate of progression of symptoms, and any symptoms of virilization (deepening of the voice, acne, irregular menstruation, clitoromegaly, malodorous sweat, etc.), as well as the medication history before the onset of hirsutism.

A complete general and systemic examination was performed, including palpation of the abdomen for any abdominal masses. Furthermore, weight, height, and BMI were recorded.

Ultrasonographic examination was performed for all participants on days 3–9 of their menstrual cycle.

None of the participants had received medication that affected hormone levels in the last three months before the study.

Hirsutism was qualified according to the Ferriman and Gallwey score. This score grades terminal hair density in nine different body areas under the effect of androgens from zero (lack of terminal hair) to four (widespread terminal hair growth), with a score of eight or more representing hirsutism.^14^ IH diagnosis was based on normal androgen hormone levels, including free testosterone, DHEAS, 17-hydroxyprogesterone (17OHP), serum cortisol, thyroid function test, LH/follicle-stimulating hormone (FSH) less than 3 IU/L, and normal serum prolactin, in addition to normal pelvic ultrasonography.

### Inclusion criteria

The study included Iraqi women with hirsutism, all of whom had normal adrenal and ovarian function

### Exclusion criteria

Patients with POCS, Cushing's syndrome, thyroid disorder, androgen-secreting ovarian or adrenal tumors, CAH, and patients with increased prolactin levels were excluded from the study.

### Data collection

For hormonal assessment, serum samples were collected after an overnight fast within 3–4 hours of waking on day two of the menstrual cycle.

Trained laboratory technicians aspirated 5 ml of blood by venipuncture. The blood was collected in a serum separating tube. The samples were centrifuged, and serum was separated from cells within 2 hours of collection.

For FSH and LH, multiple blood samples were collected 20–30 min apart, and an average hormone measurement was taken.

The hormones were tested using Mini Vidas' automated immunoassay system based on immunofluorescence assay.

To evaluate insulin resistance, we used the quantitative insulin sensitivity check index (QUICKI) and the homeostasis model assessment-insulin resistance (HOMA-IR) in both groups. HOMA-IR was calculated using the following formula: basal insulin concentration (μU/ml) × fasting blood glucose (FBG) (mg/dl)/405.^15^ The QUICKI was calculated from FBG (mg/dl) and insulin levels (μIU/ml), according to the following formula: QUICKI = 1/(logI_0_ + logG_0_).^16^

The Matsuda index provides information about insulin sensitivity in hepatic cells in addition to the peripheral tissue. The Matsuda index is calculated from blood glucose levels (mg/dl) and insulin levels (mIU/l) during fasting and throughout the oral glucose tolerance test (OGTT).^17^

(Matsuda) 10^4^/√[(G_0mg/dl_ × I_0mU/ml_) × (G_mean_ × I_mean_)]

where:

I_0_ – basal serum insulin (mIU/l),

G_0_ – FBG (mg/dl),

G_mean_ – mean of glucose levels throughout the OGTT (mg/dl),

I_mean_ – mean of insulin levels throughout the OGTT (mU/L),

The OGTT was taken in the early morning between 08:00 a.m. and 10:00 a.m. after 10–12 hours of fasting. A basal blood sample was collected before oral administration of 75 g of glucose, and blood samples were then collected at 30 min intervals for 2 h to determine glucose and insulin concentrations.^18^

### Statistical analysis

The collected data were tested before computerized data recording. The SPSS (Statistical Package for Social Sciences) was used for the statistical analysis of the data. The data are descriptive as mean and standard deviation. The results of both groups were compared using an unpaired *t-test*. A *p* value of ≤ 0.05 was considered significant.

## RESULTS

### Participants

We studied 60 Iraqi women with IH and 60 women without hirsutism as a control group.

### Demographic data

Baseline characteristics of patients and healthy controls are shown in Table 1. The mean age of the control group was 27.71 ± 8.2 years, while the mean age of the patients with IH was 28.31 ± 5.3 years. The BMI of the control group and the patient group was 26.6 ± 8.6 kg/m^2^ and 28.41 ± 1.1 kg/m^2^, respectively.

The mean weight of the control group and the patient group was 76.31 ± 8.7 and 78.1 ± 2.6, respectively.

### Outcome data

In the IH group, the mean FSH level was 7.1 ± 0.9 IU/L, while in the control group, it was 6.9 ± 0.3 IU/L (*p* = 0.1). The mean LH of the IH group was 5.2 ± 1.5 IU/L, while that of the control group was 5.45 ± 0.6 IU/L (*p* = 0.2). The mean DHEAS of the IH group was 1151 ± 1.8 ng/ml, while that of the control group was 1149 ± 8.6 ng/ml (*p* = 0.08). The mean 17OHP of the control group was 1.5 ± 3.4 ng/ml, while that of the IH group was 1.6 ± 6.2 ng/ml (*p* = 0.91). Prolactin levels were higher in the IH group (25.2 ± 6.5 ng/ml) than in the control group (21.8 ± 1.3 ng/ml), with a significant difference of *p* < 0.0001. There was a significant difference in free testosterone levels in the IH group (2.8 ± 5.3 pg/ml) and the control group (0.6 ± 1.4 pg/ml). Basal insulin was higher in the IH group (9.5 ± 0.2 mIU/L) than in the control group (4.8 ± 3.7 mIU/L), with a *p* value of < 0.0001, as shown in Table 2.

### Main outcomes

No significant difference was observed in the FBG levels of the two groups. The mean fasting serum glucose value of the IH group was 80.8 ± 1.1 mg/dl, while that of the control group was 79.2 ± 8.6 mg/dl (*p* = 0.15). HOMA-IR was higher in the IH group (2.3 ± 4.1 μU mg) than in the control group (0.8 ± 1.2 μU mg), with a *p* value of 0.007. The QUICKI showed a significant difference with a *p* value less than 0.0001, with the IH group having a lower QUICKI (0.31 ± 0.2 μU^-1^/mg) than the control group (0.45 ± 0.1 μU^-1^/mg). The insulin sensitivity index (ISI) (Matsuda) was significantly lower in the IH group (3.1 ± 0.4) than in the control group (7.8 ± 1.3), with a *p* value of < 0.0001, as shown in Table 3.

## DISCUSSION

Insulin is a peptide hormone secreted by the pancreatic beta cells. A high glucose concentration is the primary stimulant of insulin secretion.^19^ Insulin resistance is the reduced responsiveness of a target organ to the insulin to which it is exposed.^20^ Hirsutism is a common endocrine problem resulting from elevated androgen levels or increased sensitivity to the androgen. PCOS is considered the most common etiology of hirsutism, and there is a well-established association between PCOS and insulin resistance.^21^ However, limited studies have investigated the relationship between IH and insulin resistance. There is no clear explanation for the pathogenesis of IH. The pathophysiology of IH is currently thought to be due to increased activity of the 5α-reductase enzyme in the skin and probably to a modification in androgen receptor functions.^11^

HEC (hyperinsulinemic euglycemic clamp) is the “gold standard” test for evaluating insulin resistance.^22^ However, because the test costs money and time, simpler methods for assessing insulin sensitivity have been developed.

These methods are classified into two groups: (1) indices calculated using fasting insulin, glucose, and triglycerides and (2) indices calculated using glucose and insulin concentrations during 120 min of OGTT.

In this study, we used HOMA-IR to measure insulin resistance from basal (fasting) insulin and glucose concentrations. Another index we used in this study is the QUICKI, a variant of HOMA-IR equations, as it converts the data by taking both the logarithm and the reciprocal of the insulin–glucose product.

Finally, we used the Matsuda index, an insulin sensitivity assessment, that allows estimation of peripheral tissue and hepatic insulin sensitivity from insulin and glucose concentrations during fasting and throughout the OGTT.

In the present study, there was a significant difference in basal insulin and insulin sensitivity indices between the IH group and the control group. The HOMA-IR index was higher in patients with IH than in the control group, while the QUICKI and Matsuda index were significantly lower in the IH group than in the control subjects.

This finding is consistent with the results of other studies. Unlühizarci et al. found that women with hirsutism have some insulin resistance. They concluded that hirsute women have glucose intolerance and insulin resistance, regardless of the cause of hirsutism, whether PCOS or IH.^23^ Ucak et al., similar to our results, found that non-obese IH patients also had insulin resistance.^24^ Talaei et al. concluded that insulin resistance and hyperinsulinemia are associated with hirsutism in both PCOS and IH.^25^ Similarly, Fattah and Darwish found that basal insulin and HOMA-IR showed a significant difference between PCOS, IH, and control groups.^26^

The role of insulin resistance in IH is unclear. One of the explanations is the stimulatory effect of insulin on hair follicle growth through the insulin-like growth factor (IGF) system. Philpott et al. showed that physiological concentrations of IGF-II are potent stimulants of hair follicles in vitro, whereas insulin stimulates hair follicles in supraphysiological concentrations.^27^ Furthermore, Weger and Schlake found that IGF-1 has a significant mitogenic effect on hair follicles in mice.^28^

In contrast, Cebeci et al. did not find a significant difference in insulin levels and insulin resistance in the IH group and the control group.^29^ This discrepancy could be due to the small sample size. In addition, Raygor et al.^30^ stated that race and ethnicity may influence insulin resistance in different study populations.

## STRENGTHS AND LIMITATIONS

There are two common methods for evaluating insulin resistance: 1) the glucose clamp technique measures the rate of glucose utilization and production during steady-state hyperinsulinemia; 2) the minimal model approach measures the changes in glucose disappearance rates following an intravenous glucose challenge that are mediated by insulin. These methods lead to precise quantitative evaluations of insulin resistance. They are also quite expensive, challenging to perform, and invasive.^31^

To overcome this difficulty, several insulin resistance indices based on fasting serum samples or samples obtained during the OGTT have been developed.

The strength of this study lies in the use of multiple surrogates to estimate insulin resistance. Specifically, we used HOMA-IR, which has robust phenotypic, environmental, and genetic correlations with minimal model-based insulin sensitivity.^32^ We also used the QUICKI, a minimally invasive, accurate test for insulin sensitivity. Additionally, we used the Matsuda index and obtained several insulin measurements from OGTTs to further assess hepatic and peripheral insulin sensitivity.

However, a major limitation of this study is that it only examined Iraqi Arab women with IH in a single city (Alnajaf, Iraq). Although Arabs are the most common ethnic group in Iraq, there are many other ethnicities such as Kurds, Turkmens, Yazidis, and Assyrians. This study could be the basis for further studies evaluating insulin resistance in Iraqi women with IH of different ethnic origins and encompassing all Iraqi cities.

## CONCLUSION

The study of insulin resistance in Iraqi women with IH revealed some degree of insulin resistance and increased basal serum insulin levels. Further research with larger sample sizes is required to determine the etiology of IH. The study of the relationship between skin 5α-reductase activity and insulin resistance could be a good start. In addition, a clinical study is required to assess the impact of insulin sensitizer medication on the management of IH in Iraqi women.

### Acknowledgment

The authors would like to express their heartfelt gratitude to everyone who contributed to the completion of this project.

### Conflict of interest

The authors declare that there is no conflict of interest.

### List of abbreviations

[Table tbl4]

## Figures and Tables

**Table 1:** Basic characteristics of the two groups (IH 60 and control 60).

Variable	IH 60	Control 60	*p*

Age (years)	28.31 ± 5.3	27.71 ± 8.2	0.63

Weight (kg)	78.1 ± 2.6	76.31 ± 8.7	0.12

BMI (kg/m^2^)	28.41 ± 1.1	26. 6 ± 8.6	0.10


**Table 2:** Baseline hormonal parameters in the two groups (IH 60 and control 60).

Variable	IH	Control	*p*

FSH (IU/L)	7.1 ± 0.9	6.9 ± 0.3	0.1

LH (IU/L)	5.2 ± 1.5	5.45 ± 0.6	0.2

DHEAS (ng/ml)	1151 ± 1.8	1149 ± 8.6	0.08

Prolactin (ng/ml)	25.2 ± 6.5	21.8 ± 1.3	< 0.0001

17OHP (ng/ml)	1.6 ± 6.2	1.5 ± 3.4	0.91

Free testosterone (pg/ml)	2.8 ± 5.3	0.6 ± 1.4	0.002

Insulin (mIU/L)	9.5 ± 0.2	4.8 ± 3.7	< 0.0001


FSH: follicular phase (1.37–9.9 IU/L), LH: follicular phase (1.6–15 IU/L), DHEAS: 900–3600 ng/ml), prolactin: 3–27 ng/ml, 17OHP: 0.35–2.90 ng/ml, free testosterone: 0–4.2 pg/ml, insulin: < 25 mIU/L.

**Table 3:** Parameters of insulin resistance in IH 60 and control subjects 60.

Variable	IH	Control	*p*

FBG (mg/dl)	80. 8 ± 1.1	79.2 ± 8.6	0.15

HOMA-IR (7μU mg)	2.3 ± 4.1	0.8 ± 1.2	0.007

QUICKI (Uμ^-1^/mg)	0.31 ± 0.2	0.45 ± 0.1	< 0.0001

ISI (Matsuda)	3.1 ± 0.4	7.8 ± 1.3	< 0.0001


FBG: fasting blood glucose (76–100 mg/dl), HOMA-IR: < 1 optimum insulin sensitivity, >1.9 initial insulin resistance, >2.9 significant insulin resistance, QUICKI: a value less than 0.339 μU^-1^/mg indicates insulin resistance, ISI: insulin sensitivity index (Matsuda) < 4.5 indicates insulin resistance.

**Table tbl4:** 

CAH	Congenital Adrenal Hyperplasia

DHEAS	Dehydroepiandrosterone Sulfate

FBG	Fasting Blood Glucose

FSH	Follicle-Stimulating Hormone

HEC	Hyperinsulinemic Euglycemic Clamp

HOMA-IR	Homeostasis Model Assessment-Insulin Resistance

IH	Idiopathic Hirsutism

ISI	Insulin Sensitivity Index

LH	Luteinizing Hormone

OGTT	Oral Glucose Tolerance Test

17OHP	17-Hydroxyprogesterone

PCOS	Polycystic Ovarian Syndrome

SHBG	Sex Hormone-Binding Globulin

SPSS	Statistical Package for Social Sciences

